# The impact of online and offline social support on the quality of life of HIV/AIDS patients: a cross-sectional study

**DOI:** 10.3389/fpubh.2025.1688797

**Published:** 2025-12-03

**Authors:** Zhiwei Zhou, Chuanwu Sun, Feifei Chai, Hui Zhou, Chengzhong Wang, Yaodong Zhang

**Affiliations:** 1 Xuzhou Center for Disease Control and Prevention, Xuzhou, Jiangsu, China; 2 Sixian People's Hospital, Suzhou, Anhui, China; 3 Xuzhou Medical University, Xuzhou, Jiangsu, China

**Keywords:** cross-sectional study, HIV/AIDS, mediation analysis, offline social support, online social support, quality of life

## Abstract

**Background:**

Online and offline social support may shape the quality of life (QoL) of people living with HIV/AIDS (PLWHA), yet their relative and joint contributions remain unclear.

**Methods:**

We conducted a cross-sectional survey of 605 PLWHA in Xuzhou, gathering demographic data, subjective well-being, socioeconomic status, HIV/AIDS knowledge, offline and online social support, and QoL. Correlation analysis, multiple linear regression, and mediation-moderation models quantified associations.

**Results:**

Higher socioeconomic status, greater subjective well-being, richer HIV/AIDS knowledge, and stronger offline and online support independently predicted better QoL (all *p* < 0.05). Offline support showed a direct effect on QoL (β = 0.14, *p* < 0.001) and an indirect effect via online support (indirect β = 0.024, *p* < 0.05). Online support also improved QoL (β = 0.15, *p* < 0.001) but did not moderate the offline-QoL link (interaction β = 0.006, *p* > 0.05).

**Conclusion:**

Offline support boosts QoL both directly and by fostering online support, whereas online support alone confers additional benefit without buffering deficits in offline support. Integrating face-to-face and digital support is therefore crucial for optimising patient outcomes.

## 1 Introduction

Since the late twentieth century, HIV/AIDS has emerged as a critical global public-health issue. Since its initial outbreak, the epidemic has resulted in more than 84 million infections and over 40 million deaths worldwide. In China, AIDS-related mortality exceeds the combined total of deaths from all other infectious diseases, highlighting the gravity of the situation and the need for further research (1). With the widespread availability of ART, research priorities have increasingly emphasised health-related quality of life (QoL) (2–4).

Social support is a pivotal determinant of quality of life; it refers to the network through which individuals perceive care, respect and assistance from others (5). It encompasses multiple forms, including emotional, instrumental, informational and appraisal support (6, 7). Adequate social support is consistently associated with better mental health and higher overall quality of life, and it can, to some extent, buffer the adverse effects of HIV-related stigma across settings (8, 9). Evidence further suggests that robust social support benefits physical and mental health and may enhance immune function (10).

With the rapid advancement of information technology, social support has expanded beyond offline contexts to include online modalities. Offline support primarily comprises in-person interactions with family members, friends, community services and psychological counselling, providing direct emotional and material assistance (11).

Online social support—delivered through social media, virtual support groups and e-health platforms—provides emotional and informational assistance via virtual interactions. In today’s digital age, a growing number of individuals turn to the internet for help. On the one hand, online support transcends geographical and temporal barriers, offering convenient channels for individuals in remote areas or with limited mobility, thereby complementing traditional face-to-face services (12). On the other hand, the anonymity and privacy afforded by the internet reduce the psychological threshold for seeking help without publicly disclosing one’s identity (13).

In the antiretroviral therapy (ART) era, improving health-related quality of life (QoL)—a multidimensional construct spanning physical, psychological, social, environmental, independence, and spiritual domains—has become a key objective of HIV care (3, 14, 15). Building on this shift, the global HIV agenda has moved beyond viral suppression towards the proposed “fourth 90,” namely that the vast majority of people living with HIV/AIDS (PLWHA) who achieve viral suppression should also report good QoL (2). Yet even under effective ART, non-AIDS comorbidities, persistent stigma, and broader social determinants continue to depress QoL across multiple domains, underscoring the need to address psychosocial as well as biomedical needs (3, 4).

Among modifiable determinants, social support is consistently associated with better QoL in PLWHA by buffering stress and stigma, enhancing self-efficacy, and facilitating engagement in care (5, 6, 8, 10). In today’s support ecology, both offline (family, peers, community, and clinical services) and online modalities (social media, virtual peer groups, and e-health platforms) are salient. Evidence from China indicates that digital interventions (e.g., WeChat-based programmes) can reduce depressive symptoms and improve psychosocial adaptation—suggesting that online support can complement rather than replace face-to-face services; however, over-reliance on internet-based ties may carry risks such as problematic use (16–18). Optimal care therefore requires integrating online with offline support to achieve sustained QoL gains. Nonetheless, few studies have concurrently modelled both modalities and formally tested mediation and moderation, and evidence from China remains limited.

Context also matters. Much of the extant literature derives from Western settings and may not capture features salient to non-Western contexts—such as the pivotal role of family networks within China’s collectivist culture, where family-based care and stigma processes shape help-seeking and perceived support (19). Understanding how online and offline support jointly operate in such contexts is essential for designing QoL-oriented, culturally responsive interventions.

Accordingly, we conducted a cross-sectional survey to quantify the independent and joint associations of offline and online social support with QoL among PLWHA. We hypothesised that offline support would be directly associated with higher QoL and would also exert an indirect effect by strengthening individuals’ online support (i.e., partial mediation). In addition, we tested whether online support moderates the offline support–QoL association.

By addressing these questions, our findings aim to inform public-health practise and promote the integration of online and offline support systems, thereby more effectively meeting the diverse support needs of people living with HIV.

## 2 Research methods

### 2.1 Study subjects

Xuzhou HIV/AIDS case records between 1 January 2018 and 31 December 2023 were identified from the web-based case reporting system of the Chinese Center for Disease Control and Prevention (China CDC). After restricting to individuals aged 18–70 years who were alive at survey, 1,406 eligible records constituted the sampling frame (out of 1,709 newly reported during 2018–2023). We then implemented a stratified random sampling design with equal allocation by county/district and calendar year (11 counties/districts × 6 years = 66 strata). Within each stratum, participants were selected without replacement using computer-generated random numbers from de-duplicated lists: 10 per year in each county/district, except Jiawang District and the Economic Development Zone, where 5 per year were drawn by design. Where a stratum had fewer eligible individuals than its target, all eligible individuals were invited. A small random reserve list was prepared in each stratum for replacements in case of non-response or ineligibility. The target sample size was 600; the final analytic sample comprised 605 participants.

Inclusion criteria were: (1) age ≥ 18 years at diagnosis; (2) current residence recorded on the case report form as being within Xuzhou jurisdiction; (3) no mental disorders, intellectual disabilities or consciousness impairments that could affect data collection (as determined from case records and clinician assessment); and (4) signed informed consent noted in the case files.

Exclusion criteria were: (1) cases recorded as deceased in the system; (2) cases with missing key information or lost to follow-up; and (3) individuals who did not meet any of the above inclusion criteria.

After independent verification by two reviewers and ethical approval, a total of 605 eligible participants were included in the final analysis.

### 2.2 Methods

#### 2.2.1 Demographic data

A pre-tested and validated questionnaire was employed to collect sociodemographic characteristics—including age, sex, educational level, household income, marital status and occupation—as well as information on infection stage, transmission route and HIV-related knowledge. Following informed consent, trained interviewers collected data through individual face-to-face interviews.

#### 2.2.2 Offline social support

Offline social support refers to the emotional, material and informational assistance that individuals receive from family members, friends, community services and psychological counselling when facing life challenges. Emotional support comprises comfort, understanding and care; material support comprises financial aid and tangible resources; informational support encompasses advice, guidance and essential daily information. Collectively, these forms of support facilitate help-seeking, alleviate stress and enhance individuals’ capacity to cope with difficulties.

Offline social support was assessed with the Social Support Rating Scale (SSRS) developed by Xiao (20). The SSRS comprises 10 items across three dimensions—subjective support (4 items), objective support (3 items), and support utilisation (3 items). Representative items cover perceived closeness of community ties (e.g., relationships with neighbours), sources of practical help in urgent situations, and the tendency to seek help when troubled. Items are rated on 4-point Likert-type scales or, for items assessing available sources, by counting the number of sources and recoding into four categories. The three subscales have theoretical maxima of 32 (subjective support), 22 (objective support), and 12 (support utilisation), yielding a total score range of 12–66. Higher scores indicate stronger social support.

Prior Chinese studies have reported solid psychometrics for the SSRS, including excellent two-month test–retest reliability (≈0.92) and generally good internal consistency (α typically ≥0.80), alongside supportive construct validity (21, 22). In our sample (N = 605), internal consistency was good for the total scale (Cronbach’s α = 0.90) and acceptable for subscales (α = 0.81 subjective; 0.76 objective; 0.68 utilisation); corrected item–total correlations ranged 0.54–0.76.

#### 2.2.3 Online social support

Online social support refers to the emotional, informational, interactive and instrumental support that individuals obtain through internet-based platforms such as social media, online support groups and e-health services.

Online social support was assessed with the Online Social Support Scale (OSSS) developed by Liang et al. (23). The OSSS comprises 23 items across four dimensions—emotional support (8 items), informational support (5 items), instrumental support (5 items), and social-membership/companionship support (5 items)—rated on a 5-point Likert scale from 1 (strongly disagree) to 5 (strongly agree); total scores range from 23 to 115, with higher scores indicating greater online social support. Representative items include opportunities to confide feelings when lonely (emotional support), receiving advice or information online (informational support), obtaining material or service help via the Internet (instrumental support), and perceiving a sense of belonging in online groups (social-membership/companionship support).

Prior studies in Chinese samples report sound psychometric properties. The development paper supported the four-factor structure via exploratory and confirmatory factor analysis and found acceptable internal consistency and test–retest reliability (Cronbach’s α and *r* ≥ 0.70) (23), and subsequent applications in college students reported high internal consistency (e.g., total α = 0.94, subscale α = 0.77–0.89) (24, 25).

In the present study, the OSSS showed excellent internal consistency (Cronbach’s α = 0.96).

#### 2.2.4 Quality of life

The WHOQOL-HIV-BREF (developed in 2004 by the WHOQOL-HIV Group and further validated in 2012 by O’Connell et al.) consists of 31 items covering six domains: physical health, psychological health, level of independence, social relationships, environment, and spiritual/religious/personal beliefs, along with two general items assessing overall quality of life (14, 26). Each item is rated on a five-point Likert scale. Negatively worded items (e.g., pain or negative feelings) were reverse-coded. Domain scores were computed as the mean of items within each domain multiplied by four (range: 4–20), with higher scores indicating better quality of life.

Example items from each domain include: “To what extent does pain interfere with your daily life?” (physical), “Do you feel your life has meaning?” (psychological), “How well are you able to concentrate?” (independence), “To what extent are you bothered by others blaming you for your HIV status?” (social), “How safe do you feel in your daily life?” (environment), and “How often do you experience negative feelings?” (spiritual). In this study, the WHOQOL-HIV-BREF demonstrated excellent internal consistency, with a Cronbach’s α coefficient of 0.903 across the six domains, supporting its reliability for assessing quality of life among individuals living with HIV/AIDS.

### 2.3 Quality control

Before data collection, a standardised protocol was developed and all interviewers received centralised training to ensure consistent procedures and content. During the study, informed consent was obtained from all participants, and individual face-to-face interviews were conducted to protect privacy. Afterwards, data were entered into a central database and preliminarily checked for completeness and accuracy. Logical checks identified duplicate entries and missing values. Any discrepancies were resolved by re-contacting participants or interviewers to verify information, thereby ensuring data consistency and reliability.

### 2.4 Statistical methods

Data were organised in Excel 2016 and analysed using SPSS version 25.0. Continuous variables are presented as mean ± standard deviation and were compared using independent-samples *t* tests (two groups) or one-way ANOVA (multiple groups); categorical variables are reported as frequencies and percentages. Pearson correlation coefficients quantified associations among online social support, offline social support, and quality of life (QoL).

Determinants of QoL were evaluated using hierarchical multiple linear regression. Covariates were selected *a priori* based on conceptual relevance to QoL in people living with HIV. In Block 1 (Model 1), we entered socioeconomic status (SES), happiness (life satisfaction), and HIV knowledge awareness as control variables; Block 2 (Model 2) added the focal predictors (offline and online social support); for the moderation test, Block 3 (Model 3) further added the interaction term (offline × online social support). SES was dichotomised as a binary variable and entered using dummy coding (0 = lower-middle class and below, 1 = middle class and above). Other categorical predictors were entered via dummy (indicator) coding with prespecified reference groups (happiness: unhappy; HIV knowledge: unaware). Standardised beta coefficients are reported.

Clinical variables available in our dataset—disease stage (HIV-infected vs. AIDS), CD4^+^ T-cell categories (<200, 200–350, >350 cells/μL), and AIDS-related symptoms (yes/no; see Table 1)—were examined descriptively and in univariate analyses as potential confounders. All participants were receiving antiretroviral therapy (ART) at the time of the survey, and viral load data were not collected; therefore, ART status and viral load could not be included as covariates in the multivariable models. For the prespecified primary hierarchical models aimed at estimating the association between social support and QoL, we did not include clinical variables in any block to avoid potential over-adjustment (clinical status may partly lie on the pathway linking social support to QoL via care engagement/adherence). We acknowledge this choice as a study limitation.

**Table 1:** Demographic characteristics of 605 HIV/AIDS cases in Xuzhou [*n* (%)].

Characteristics		HIV/AIDS cases (*n* = 605), *n* (%)
Gender	Male	538 (88.93%)
	Female	67 (11.07%)
Ethnicity	Han	601 (99.34%)
	Minority	4 (0.66%)
Marital status	Married	280 (46.28%)
	Unmarried	325 (53.72%)
Education level	Junior high or below	238 (39.34%)
	High school or above	367 (60.66%)
Occupation	Farmer	269 (44.46%)
	Commercial service	209 (34.55%)
	Enterprise/institution	33 (5.45%)
	Retired/unemployed	94 (15.54%)
Annual household income	<30,000 CNY	256 (42.31%)
	≥30,000 CNY	349 (57.69%)
Income source	Salary	361 (59.67%)
	Non-salary^a^	244 (40.33%)
Sample source	Testing/consulting	279 (46.12%)
	Medical institution	278 (45.95%)
	Other^b^	48 (7.93%)
Transmission route	Heterosexual	240 (39.67%)
	Homosexual	363 (60.00%)
	Other^c^	2 (0.33%)
Disease stage	HIV-infected	442 (73.06%)
	AIDS patient	163 (26.94%)
CD4 + T-cell level	<200 cells/μL	86 (14.21%)
	200–350 cells/μL	117 (19.34%)
	>350 cells/μL	402 (66.45%)
AIDS-related symptoms	No	574 (94.88%)
	Yes	31 (5.12%)
Referred for treatment	Yes	100 (100.00%)
	No	0 (0.00%)
Medical insurance	No	45 (7.44%)
	Yes	560 (92.56%)
Socioeconomic status	Middle class and above	197 (32.56%)
	Lower-middle class and below	408 (67.44%)
Life satisfaction	Unhappy	71 (11.74%)
	Neutral	280 (46.28%)
	Happy	254 (41.98%)
HIV/AIDS knowledge awareness	Aware	567 (93.72%)
	Unaware	38 (6.28%)

a Non-wage income includes activities such as farming, business, payments from relatives, and relief funds; wage income includes earnings from non-agricultural sectors or off-farm employment, even for those registered as farmers.

b Voluntary blood donation testing, health checks for entertainment venue staff, testing for positive spouses or sexual partners, special surveys, etc.

c Intravenous drug use.

Mediation by online social support in the association between offline social support and QoL was tested using a non-parametric bootstrap procedure (5,000 resamples) to estimate indirect effects and bias-corrected 95% CIs. All three equations in the mediation analysis (exposure→mediator, exposure→outcome, and mediator→outcome) were adjusted for the same covariate set—SES, happiness, and HIV knowledge—to avoid differential confounding. Given the cross-sectional design, all analyses assessed associations rather than causal relationships. A two-tailed *p* < 0.05 was considered statistically significant.

### 2.5 Sample size and power

We assessed sample size adequacy *a priori*. Following Green’s rule-of-thumb for multiple regression (minimum *N* ≥ 50 + 8 m for testing the overall model, where m is the number of predictors), a sample of at least *N* ≥ 90 would be required for our final model with *m* = 5 predictors. In addition, an *a priori* power analysis was conducted in G*Power (version 3.1) (15, 27) for linear multiple regression (fixed model, R^2^ deviation from zero) assuming α = 0.05, power (1 − β) = 0.80, and a small-to-moderate effect size (*f*^2^ = 0.05) with up to 10 predictors; this yielded a required sample of approximately *N* ≈ 135. Under a more conservative small effect (*f*^2^ = 0.02), the required sample is approximately *N* ≈ 320. Our achieved sample (*N* = 605) exceeds these thresholds, indicating adequate power for the planned analyses. For mediation, the bias-corrected bootstrap with 5,000 resamples was used; given *N* = 605, this procedure provides stable confidence intervals for small indirect effects. Power calculations were based on the five predictors in the final model (*m* = 5). For completeness, even under a conservative assumption including age, sex, and education (*m* = 8), our sample (*N* = 605) still exceeds Green’s threshold (*N* ≥ 114) and the G*Power requirement for small-to-moderate effects.

### 2.6 Missing data

Item-level missingness was low (<5%) for all analysis variables. We used complete-case analysis (listwise deletion). Under a missing-at-random assumption and with low missingness, listwise deletion yields unbiased estimates. As a sensitivity check, key descriptive characteristics did not materially differ between included and excluded cases.

## 3 Results

### 3.1 Demographic characteristics and socioeconomic profile of the study participants

In the survey, 88.93% (*n* = 538) of participants were male, whereas 11.07% (*n* = 67) were female. The vast majority were Han Chinese (99.34%), with a mean age of 40.1 years. Regarding educational attainment, 60.66% had completed senior high school or higher. Marital status was divided as follows: 46.28% were married or cohabiting, while 53.72% were unmarried. In terms of occupation, 44.46% were farmers, 34.55% worked in business or service sectors, 15.54% were retired or unemployed, and 5.45% were employed in enterprises or public institutions. An annual household income exceeding 30,000 RMB was reported by 57.69%, and salary was the main income source for 59.67% of participants.

Most cases were identified through voluntary counselling and testing (46.12%) or routine screening in medical institutions (45.95%). Transmission was predominantly sexual: 60.00% via male–male sexual contact and 39.67% via heterosexual contact. Of the participants, 73.06% were at the HIV infection stage, whereas 26.94% had progressed to AIDS. A majority (66.45%) had CD4^+^ T-lymphocyte counts > 350 cells mm^−3^, and 94.88% were asymptomatic. All participants were receiving antiretroviral therapy (ART), and 92.56% had medical insurance. Regarding socioeconomic status, 67.44% were in the lower-to-middle tiers, and 41.98% reported feeling happy. Awareness of HIV-related knowledge reached 93.72%.

### 3.2 Offline social support situation

Female participants scored significantly higher than males on objective, subjective and overall offline social support (*p* < 0.05). Participants aged 18–39 years recorded lower objective, subjective and overall offline social-support scores than those aged ≥ 40 years, yet exhibited higher support-utilisation scores (*p* < 0.001). Married or cohabiting individuals reported greater objective, subjective and overall offline social support than those without a spouse or partner (*p* < 0.001). Employees of enterprises or public institutions showed significantly higher support utilisation and overall offline social-support scores than other occupational groups (both *p* < 0.001); their subjective support scores were higher than some groups but did not exceed those of farmers (see Supplementary Table S1). Higher annual household income was associated with superior objective-support and support-utilisation scores (*p* < 0.01), and participants whose income derived primarily from non-salary sources had higher overall offline social-support scores than salary earners (*p* < 0.05). Insured participants obtained higher objective, subjective and overall offline social-support scores than those without insurance (*p* < 0.05). Individuals in higher socioeconomic strata outperformed those of lower status on subjective, utilisation and overall offline social-support scores (*p* < 0.01). Finally, respondents reporting high life satisfaction achieved higher scores across all offline social-support dimensions (*p* < 0.01). Detailed results are presented in Supplementary Table S1.

### 3.3 Online social support situation

Significant differences in online social support were observed across demographic characteristics. Men scored significantly higher than women on the informational, companionship and emotional dimensions and on the overall online social-support score; the instrumental dimension did not differ significantly by sex (*p* = 0.129). Participants aged 18–39 years exhibited significantly higher scores across all support dimensions than those aged ≥ 40 years (*p* < 0.001). Individuals without a spouse or partner scored significantly higher across all support dimensions than those who were married or cohabiting (*p* < 0.05). Participants with senior-high-school education or higher recorded significantly greater online social support than those educated to junior-high-school level (*p* < 0.001).

Employees of enterprises or public institutions obtained significantly higher informational-support scores than participants in other occupations (*p* < 0.001). By contrast, workers in commercial-service sectors achieved higher companionship, emotional, instrumental and overall online social-support scores than other occupational groups (*p* < 0.05). Higher annual household income was associated with superior scores across all dimensions of online social support relative to lower-income groups (*p* < 0.01). Participants with salaried income scored significantly higher on informational support and overall online social support than those whose income was non-salaried (*p* < 0.05).

Individuals in the HIV infection stage scored significantly higher than those with AIDS on companionship, emotional and overall online social support (*p* < 0.05). Participants of higher socioeconomic status outperformed those of lower status on informational, emotional, instrumental and overall online social support (*p* < 0.05). Participants reporting high life satisfaction obtained significantly higher scores across all support dimensions than those who did not (*p* < 0.05). Finally, participants with greater HIV-related knowledge scored significantly higher in all support dimensions than those with limited awareness (*p* < 0.01). Detailed statistics are provided in Supplementary Table S2.

### 3.4 The impact of different demographic characteristics on the quality of life scores of HIV/AIDS patients

Male participants scored significantly higher than female participants in the psychological, independence and environmental domains, as well as in the overall quality-of-life score (*p* < 0.05). Participants with senior-high-school education or higher recorded significantly better scores in the physical, psychological, independence, social-relationships and environmental domains, and in overall quality of life (*p* < 0.05). Employees of enterprises or public institutions outperformed other occupational groups across all domains (*p* < 0.05). Participants with an annual household income ≥ 30,000 RMB achieved significantly higher scores in the physical, psychological, social-relationships, environmental and spiritual domains, and in overall quality of life, than low-income peers (*p* < 0.05). Salary earners likewise scored higher in several domains than non-salary earners (*p* < 0.05). Individuals in the HIV infection stage surpassed those with AIDS in the physical domain and overall score (*p* < 0.05). Participants with CD4^+^ T-lymphocyte counts > 350 cells mm^−3^, or without AIDS-related symptoms, displayed significantly better scores in multiple domains (*p* < 0.05). Participants with medical insurance scored higher than the uninsured in the spiritual/religious/personal beliefs domain (*p* = 0.047); no significant differences were observed in the other domains. Higher socioeconomic status and high life satisfaction were associated with significantly superior scores across all domains (*p* < 0.01), as was greater HIV-related knowledge (*p* < 0.05). Detailed results are provided in Supplementary Table S3.

### 3.5 Correlation analysis of online social support, offline social support, and quality of life

Both offline and online social support were positively correlated with overall quality of life and with each individual domain score. For offline social support, associations were strongest with the spiritual and psychological domains; for online social support, they were strongest with the environmental and spiritual domains. Correlation coefficients are presented in Table 2.

**Table 2:** Pearson correlation coefficients of online social support, offline social support, and quality of life (*n* = 605).

Item	Online social support	Quality of life	Physical domain	Psychological domain	Independence domain	Social relationships domain	Environmental domain	Spiritual domain
Offline social support	0.188**	0.288**	0.154**	0.260**	0.142**	0.358**	0.302**	0.197**
Online social support		0.264**	0.144**	0.190**	0.187**	0.295**	0.294**	0.192**

**P < 0.01.

### 3.6 Multiple linear regression analysis of factors influencing quality of life

A multiple linear regression model was fitted with the overall quality-of-life score as the dependent variable and socioeconomic status, subjective well-being, HIV-related knowledge, total offline social-support score and total online social-support score as predictors. Higher socioeconomic status, greater subjective well-being, better HIV-related knowledge and higher offline and online social-support scores were each independently associated with improved quality of life among individuals living with HIV/AIDS. Regression coefficients are presented in Table 3.

**Table 3:** Multivariate regression analysis of factors affecting quality of life.

Influencing factors	Unstandardised coefficient		(Beta)	*t*	*P*	Collinearity statistics	
	*B*	SE				Tolerance	VIF
Constant	44.941	2.720		16.521	<0.01		
Socioeconomic status	5.700	0.905	0.218	6.296	<0.01	0.902	1.109
Subjective well-being							
Average	5.292	1.336	0.215	3.961	<0.01	0.366	2.734
Happiness	6.264	0.703	0.505	8.915	<0.01	0.337	2.963
Awareness of HIV/AIDS	4.379	1.720	0.087	2.546	0.011	0.932	1.073
Offline social support	0.214	0.052	0.141	4.097	<0.01	0.911	1.098
Online social support	0.122	0.028	0.150	4.353	<0.01	0.913	1.096

SES was dummy-coded (1 = middle and above; 0 = lower-middle and below [reference]).

### 3.7 Moderating effect of online social support in the relationship between offline social support and quality of life

Hierarchical multiple regression indicated that the control variables—socioeconomic status, subjective well-being and HIV-related knowledge—jointly accounted for 30.6% of the variance in quality of life (Model 1: *R*^2^ = 0.306, *F* = 66.23), with subjective well-being exerting the strongest effect. Incorporating offline social support (β = 0.14) and online social support (β = 0.15) in Model 2 increased the explained variance to 35.3%, demonstrating that each form of support independently and positively predicted quality of life. The interaction term between offline and online social support entered in Model 3 was not significant (β = 0.006, *p* = 0.862), indicating that online social support did not moderate the association between offline support and quality of life. Regression estimates are summarised in Table 4.

**Table 4:** Moderating effect of online social support in the relationship between offline social support and quality of life.

Variable	Quality of life		
	Model 1	Model 2	Model 3
Constant	56.67	44.94	44.96
Control variables			
Socioeconomic status	0.24***	0.22***	0.22***
Happiness (reference: unhappy)			
Neutral	0.26***	0.22***	0.22***
Happy	0.58***	0.51***	0.51***
HIV/AIDS Awareness	0.12***	0.09**	0.09**
Independent variable			
Offline social support		0.14***	0.14***
Moderator variable			
Online social support		0.15***	0.15***
Interaction term			
Offline social support × online social support			0.006
R2	0.306	0.353	0.353
*F*	66.230***	54.268***	46.445***

** indicates *p* < 0.01, *** indicates *p* < 0.001; all regression coefficients in the table are standardised regression coefficients. SES coding as in Table 3.

### 3.8 Indirect pathway analysis of offline and online social support in relation to quality of life

Offline social support was positively correlated with online social support (β = 0.16, *p* < 0.01), and online social support, in turn, was positively correlated with quality of life (β = 0.15, *p* < 0.01). A bias-corrected bootstrap procedure comprising 5,000 resamples identified a small but significant indirect path from offline social support to quality of life via online social support (β_indirect = 0.024, 95 %CI 0.008–0.045), which accounted for 14.1% of the total effect (β_total = 0.17). The direct effect of offline social support on quality of life remained significant after inclusion of the mediator (β_direct = 0.14), indicating partial rather than full mediation (Table 5).

**Table 5:** Mediating effect of online social support on the relationship between offline social support and quality of life.

Variable	Online social support		Quality of life		
	Model 1	Model 2	Model 3	Model 4	Model 5
Constant	53.56	46.17	56.67	50.57	44.94
Control variables					
Socioeconomic status	0.07	0.06	0.24***	0.23***	0.22***
Happiness (reference: unhappy)					
Neutral	0.15*	0.13*	0.36***	0.24***	0.22***
Happy	0.17*	0.12	0.58***	0.52***	0.51***
HIV/AIDS awareness	0.19***	0.19***	0.12**	0.12**	0.09*
Independent variable					
Offline social support		0.16***		0.17***	0.14***
Mediator variable					
Online social support					0.15***
R2	0.062	0.087	0.306	0.332	0.353
*F*	9.985***	11.487***	66.230***	59.547***	54.268***

Indirect effect (offline → online → QoL): β_indirect = 0.024; bias-corrected 95% CI = 0.008–0.045 (bootstrap, 5,000 resamples); proportion mediated = 14.1% of the total effect (β_total = 0.170). **p < 0.01, ***p < 0.001. All regression coefficients are standardised β. SES coded as in Table 3.

## 4 Discussion

By investigating both offline and online social support, this study identifies disparities in the support available to individuals living with HIV/AIDS across demographic strata—gender, age, income, marital status, occupation and health-insurance coverage—and clarifies how these factors influence quality of life. The findings indicate that individuals with higher socioeconomic status, stronger subjective well-being and greater HIV-related awareness exhibit significantly better quality of life. Moreover, both online and offline social support exert significant positive effects on quality of life.

The results show that demographic differences lead to significant variation in how participants acquire and use social support. First, female participants scored higher on offline social support, potentially reflecting women’s greater willingness to express emotions and to receive care from family members and close contacts (28). By contrast, male participants excelled in online social support, suggesting that men may prefer to seek practical information on the internet when coping with challenges. Age likewise shaped support patterns: individuals aged ≥ 40 years scored higher on offline social support, perhaps owing to more stable finances and social networks, whereas younger participants obtained higher online support, reflecting their familiarity with and reliance on digital platforms (29, 30).

Marital status, occupation, income and insurance jointly shaped the way participants secured help. Married or cohabiting individuals obtained markedly stronger offline support, whereas unmarried peers relied more on the internet and scored higher across all dimensions of online support (31). Stable employment and higher earnings amplified both channels: employees of enterprises or public institutions, along with other high-income earners, recorded the highest overall support. Insured participants outperformed the uninsured on every metric, confirming that formal coverage provides an additional layer of security (32).

Disease stage adds a second fault line. Offline support does not differ significantly between HIV-stage and AIDS-stage patients, yet those still in the HIV stage receive substantially more online support. Relatively milder symptoms and greater optimism may prompt them to engage actively with virtual communities, while the heavier physical and psychological burden of AIDS can dampen online outreach. Taken together, these demographic and clinical stratifiers highlight the need for tailored interventions that match each subgroup’s specific support gaps.

The gender gap observed in this study mirrors findings from Ethiopia and India: women scored consistently lower than men in the psychological, independence and environmental domains of quality of life, underscoring the structural disadvantages they face in economic resources and social roles (33, 34).

We also found that participants who remained in the HIV infection stage, had higher CD4^+^ T-cell counts and were free of AIDS-related complications enjoyed significantly better quality of life than other groups. This observation aligns with evidence that physical health underpins psychological and social functioning, which together constitute the core of quality of life (4, 35).

Moreover, individuals with higher educational attainment, greater household income and stable earnings reported markedly better quality of life. Employment not only provides economic security but also enhances self-esteem and social support, whereas advantages in education and income facilitate access to health information and strengthen self-management (36, 37).

The findings indicate that both online and offline social support significantly enhance the quality of life among individuals living with HIV/AIDS, complementing one another in promoting well-being and mental health. Regression analysis demonstrates that increased levels of both online and offline support are associated with higher overall quality-of-life scores, underscoring the importance of these two complementary forms of support. Online social support provides multidimensional benefits to individuals living with HIV/AIDS. One study based on social media platforms reports that reducing stigma, providing a safe space, and strengthening perceived support are closely linked to sustained engagement with these platforms (38). Online support offers timely medical information and psychosocial resources that assist individuals in managing their condition and adapting psychologically. Other studies have shown that individuals living with HIV can obtain substantial online social support through microblog communities, which is strongly correlated with offline support; in fact, online support can partially compensate for deficits in offline support (39), factors such as interaction frequency and the reciprocity of exchanges can influence the effectiveness of online social support (40). These findings are consistent with our results, highlighting how online communities can transcend geographical and social barriers by offering diverse forms of support, including practical advice, emotional encouragement and peer experience-sharing.

While online communities provide valuable benefits, they also entail risks of interpersonal conflict and excessive self-disclosure, potentially leading to psychological distress (41) and managing online support communities must therefore balance platform oversight with the protection of user privacy, maximising positive effects while minimising potential harms.

The present study corroborates earlier work in showing that both offline and online social support positively affect quality of life (42), we found no evidence that online social support moderates the association between offline support and quality of life. This outcome may stem from online and offline support influencing quality of life through distinct pathways, thereby yielding no significant interaction.

Because offline support exerts a direct effect and online support transmits part of this effect indirectly, integrating the two resources into a synergistic system may more effectively enhance the quality of life of people living with HIV. As this study is cross-sectional, these mechanistic interpretations should be verified in longitudinal or intervention research.

Building on these findings, several measures may enhance the social-support system for people living with HIV. First, online platforms should be further optimised to increase functionality and ensure high-quality information dissemination, thereby providing convenient, accurate and diverse resources. Second, family-, community- and society-based offline support must be reinforced to establish a multifaceted framework. Emphasis on online versus offline modalities should be tailored: middle-aged and older adults may rely more on community engagement and family resources, whereas younger individuals can exploit digital platforms for broader connections. Governments and civil-society organisations should intensify public education and expand dissemination of HIV-related knowledge. Collaboration among medical institutions, governmental agencies and social organisations is needed to develop network-based platforms offering integrated services—medical information, psychological counselling and online social networks—to mitigate isolation and improve quality of life.

This study has several limitations. First, the sample was drawn exclusively from Xuzhou, which limits representativeness and warrants caution when generalising the findings. Second, although we examined both online and offline social support, the study did not systematically delineate how these two forms of support interact to enhance quality of life (QoL). Third, we did not collect information on years living with HIV (e.g., date of diagnosis); because infection duration may be associated with both social support and QoL, omitting it could introduce residual confounding and potentially bias effect estimates. Fourth, we did not measure HIV-related stigma (perceived or internalised), a psychosocial factor known to correlate with lower social support and poorer QoL; failure to adjust for stigma may likewise bias our results. Fifth, in our prespecified primary hierarchical models we did not adjust for clinical severity indicators (HIV disease stage, CD4^+^ T-lymphocyte count categories, AIDS-related symptoms) to avoid potential over-adjustment; however, this choice may allow residual confounding. In addition, ART status was uniform (all participants were on ART) and viral load was not collected, which precluded their inclusion as covariates and further limited control for disease severity. Future research should broaden geographic scope and sample diversity to improve generalisability; incorporate validated stigma scales (e.g., Berger HIV Stigma Scale/short forms) and infection-duration measures to strengthen confounder control; include clinical severity and virological indicators (e.g., viral load) in multivariable models and prespecified sensitivity analyses; and adopt longitudinal or large-scale designs—ideally testing interaction and moderated-mediation models—to clarify the synergistic mechanisms and long-term effects of integrated online–offline support.

In conclusion, this integrated examination of online and offline social support underscores the importance of a multidimensional system and offers guidance for intervention and policy. Future efforts should prioritise optimising online platforms and strengthening family-, community- and society-based networks, tailored to the needs of different age groups. Greater collaboration among governmental agencies, medical institutions and social organisations is required to integrate medical information, psychological counselling and social-engagement services, thereby reducing isolation, promoting well-being and ultimately enhancing quality of life.

## Data Availability

The original contributions presented in the study are included in the article/Supplementary material, further inquiries can be directed to the corresponding author.
