# Supplementary material for: The impact of online and offline social support on the quality of life of HIV/AIDS patients: a cross-sectional study
Source: Front Public Health. 2025 Dec 3;13:1688797. doi: 10.3389/fpubh.2025.1688797 (PMC12708325; doi:10.3389/fpubh.2025.1688797)
Supplement: Supplementary file 1 [file Table_1.DOCX]

Supplementary Material ：

Table S1 Social support scores of the 605 survey participants

Table S2 Domain scores of online social support stratified by demographic characteristics

Table S3 Analysis of the Impact of Different Variable Characteristics on the Quality of Life Scores of HIV/AIDS Patients in Various Domains

Table S1. Social Support Scores of Survey Participants (Mean ± SD)

| Variable | Objective Support | Subjective Support | Support Utilisation | Social Support |
| --- | --- | --- | --- | --- |
| Gender |  |  |  |  |
| Male (n=538) | 5.52$\pm$2.75 | 17.15$\pm$5.19 | 5.67$\pm$1.84 | 28.33$\pm$7.96 |
| Female (n=67) | 6.76$\pm$2.44 | 18.78$\pm$5.90 | 5.58$\pm$1.90 | 31.12$\pm$8.65 |
| t | 12.513 | 4.656 | 0.139 | 7.147 |
| P | <0.001 | 0.034 | 0.710 | 0.008 |
| Age (years) |  |  |  |  |
| 18–39 (n=337) | 5.28$\pm$2.74 | 16.35$\pm$5.00 | 5.77$\pm$1.87 | 27.40$\pm$8.01 |
| ≥40 (n=268) | 6.13$\pm$2.68 | 18.59$\pm$5.41 | 5.52$\pm$1.81 | 30.21$\pm$7.92 |
| t | 14.605 | 27.035 | 2.738 | 18.500 |
| P | <0.001 | <0.001 | <0.001 | <0.001 |
| Marital Status |  |  |  |  |
| Married (n=280) | 6.53$\pm$2.62 | 19.41$\pm$5.26 | 5.62$\pm$1.78 | 31.55$\pm$7.71 |
| Unmarried (n=325) | 4.90$\pm$2.63 | 15.54$\pm$4.63 | 5.70$\pm$1.90 | 26.14$\pm$7.55 |
| t | 57.469 | 92.665 | 0.288 | 75.793 |
| P | <0.001 | <0.001 | 0.592 | <0.001 |
| Education Level |  |  |  |  |
| Junior high or below (n=238) | 5.83$\pm$2.80 | 17.94$\pm$5.36 | 5.37$\pm$1.80 | 29.13$\pm$8.24 |
| High school or above (n=367) | 5.54$\pm$2.70 | 16.93$\pm$5.22 | 5.85$\pm$1.85 | 28.32$\pm$7.98 |
| t | 1.567 | 5.241 | 9.964 | 1.452 |
| P | 0.211 | 0.022 | 0.002 | 0.229 |
| Occupation |  |  |  |  |
| Farmer (n=269) | 5.88$\pm$2.79 | 18.43$\pm$5.11 | 5.54$\pm$1.74 | 29.84$\pm$7.72 |
| Commercial service (n=209) | 5.26$\pm$2.78 | 16.25$\pm$5.39 | 5.58$\pm$1.80 | 27.10$\pm$8.20 |
| Enterprise/Institution employee (n=33) | 5.85$\pm$3.08 | 17.70$\pm$5.96 | 6.64$\pm$2.10 | 30.18$\pm$9.70 |
| Retired/Unemployed (n=94) | 5.82$\pm$2.31 | 16.43$\pm$4.75 | 5.85$\pm$2.05 | 28.10$\pm$7.69 |
| F | 2.193 | 7.996 | 4.017 | 5.165 |
| P | 0.088 | <0.001 | <0.001 | <0.001 |
| Annual Household Income |  |  |  |  |
| <30,000 CNY (n=256) | 5.28$\pm$2.73 | 17.57$\pm$6.00 | 5.38$\pm$2.05 | 28.23$\pm$9.02 |
| ≥30,000 CNY (n=349) | 5.93$\pm$2.72 | 17.15$\pm$4.71 | 5.87$\pm$1.65 | 28.95$\pm$7.32 |
| t | 8.507 | 0.935 | 10.288 | 1.157 |
| P | 0.004 | 0.334 | 0.001 | 0.283 |
| Income Source |  |  |  |  |
| Salary (n=361) | 5.42$\pm$2.59 | 16.53$\pm$4.95 | 5.69$\pm$1.80 | 27.64$\pm$7.74 |
| Non-salary (n=244) | 6.01$\pm$2.92 | 18.50$\pm$5.58 | 5.62$\pm$1.90 | 30.13$\pm$8.35 |
| t | 6.872 | 20.722 | 0.176 | 14.168 |
| P | 0.009 | <0.001 | 0.675 | <0.001 |
| Disease Stage |  |  |  |  |
| HIV (n=442) | 5.65$\pm$2.80 | 17.41$\pm$5.26 | 5.69$\pm$1.82 | 28.75$\pm$8.05 |
| AIDS (n=163) | 5.67$\pm$2.60 | 17.09$\pm$5.40 | 5.60$\pm$1.91 | 28.36$\pm$8.19 |
| t | 0.012 | 0.440 | 0.287 | 0.269 |
| P | 0.912 | 0.507 | 0.593 | 0.604 |
| Medical Insurance |  |  |  |  |
| No (n=45) | 3.98$\pm$2.53 | 15.42$\pm$5.19 | 5.40$\pm$1.91 | 24.80$\pm$7.37 |
| Yes (n=560) | 5.79$\pm$2.71 | 17.48$\pm$5.28 | 5.68$\pm$1.84 | 28.95$\pm$8.06 |
| t | 18.723 | 6.351 | 0.977 | 11.178 |
| P | <0.001 | 0.012 | 0.323 | 0.01 |
| Socioeconomic Status |  |  |  |  |
| Lower-middle class and below (n=408) | 5.61$\pm$2.66 | 16.91$\pm$5.17 | 5.48$\pm$1.77 | 28.00$\pm$7.81 |
| Middle class and above (n=197) | 5.75$\pm$2.91 | 18.19$\pm$5.45 | 6.03$\pm$1.94 | 29.97$\pm$8.49 |
| t | 0.363 | 7.806 | 11.954 | 7.962 |
| P | 0.547 | 0.005 | 0.001 | 0.005 |
| Life Satisfaction |  |  |  |  |
| Unhappy (n=71) | 4.46$\pm$2.85 | 15.82$\pm$5.49 | 5.04$\pm$2.09 | 25.32$\pm$8.70 |
| Neutral (n=280) | 5.49$\pm$2.64 | 16.57$\pm$4.51 | 5.39$\pm$1.54 | 27.46$\pm$7.21 |
| Happy (n=254) | 6.17$\pm$2.71 | 18.58$\pm$5.77 | 6.13$\pm$1.96 | 30.88$\pm$8.24 |
| t | 12.006 | 13.338 | 16.196 | 19.885 |
| P | <0.001 | <0.001 | <0.001 | <0.001 |
| HIV/AIDS Knowledge Awareness |  |  |  |  |
| Unaware (n=38) | 4.26$\pm$2.66 | 18.37$\pm$6.13 | 4.68$\pm$1.68 | 27.31$\pm$8.36 |
| Aware (n=567) | 5.75$\pm$2.72 | 17.26$\pm$5.23 | 5.73$\pm$1.84 | 28.73$\pm$8.06 |
| t | 10.612 | 1.570 | 11.605 | 1.094 |
| P | 0.001 | 0.211 | 0.01 | 0.296 |

Table S2. Domain Scores of Online Social Support among HIV/AIDS Patients by Demographic Characteristics($\bar{x}\pm s$)

| Variable | Informational Support | Companionship Support | Emotional Support | Instrumental Support | Online Social Support |
| --- | --- | --- | --- | --- | --- |
| Gender |  |  |  |  |  |
| Male (n=538) | 17.67$\pm$3.72 | 24.61$\pm$6.70 | 18.18$\pm$4.60 | 11.82$\pm$3.20 | 72.28$\pm$14.90 |
| Female (n=67) | 15.99$\pm$3.73 | 21.82$\pm$6.15 | 16.97$\pm$4.48 | 11.19$\pm$3.18 | 65.97$\pm$15.32 |
| t | 12.201 | 10.506 | 4.168 | 2.314 | 10.633 |
| P | 0.001 | 0.001 | 0.042 | 0.129 | 0.001 |
| Age (years) |  |  |  |  |  |
| 18–39 (n=337) | 18.29$\pm$3.50 | 25.37$\pm$6.69 | 18.79$\pm$4.62 | 12.31$\pm$3.17 | 74.75$\pm$14.38 |
| ≥40 (n=268) | 16.47$\pm$3.82 | 22.96$\pm$6.46 | 17.12$\pm$4.14 | 11.06$\pm$3.11 | 67.60$\pm$14.98 |
| t | 37.303 | 20.059 | 20.121 | 23.515 | 35.510 |
| P | <0.001 | <0.001 | <0.001 | <0.001 | <0.001 |
| Marital Status |  |  |  |  |  |
| Married (n=280) | 16.84$\pm$3.39 | 23.46$\pm$6.47 | 17.44$\pm$4.34 | 11.09$\pm$3.18 | 68.83$\pm$14.78 |
| Unmarried (n=325) | 18.03$\pm$3.72 | 25.03$\pm$6.81 | 18.57$\pm$4.76 | 12.32$\pm$3.11 | 73.96$\pm$14.93 |
| t | 15.619 | 8.383 | 9.185 | 23.083 | 17.892 |
| P | <0.001 | 0.004 | 0.003 | <0.001 | <0.001 |
| Education Level |  |  |  |  |  |
| Junior high or below (n=238) | 16.34$\pm$4.00 | 22.87$\pm$6.67 | 16.95$\pm$4.59 | 11.06$\pm$3.19 | 67.22$\pm$15.56 |
| High school or above (n=367) | 18.22$\pm$3.39 | 25.23$\pm$6.55 | 18.76$\pm$4.47 | 12.20$\pm$3.13 | 74.41$\pm$14.05 |
| t | 38.221 | 18.566 | 23.064 | 19.088 | 34.736 |
| P | <0.001 | <0.001 | <0.001 | <0.001 | <0.001 |
| Occupation |  |  |  |  |  |
| Farmer (n=269) | 16.75$\pm$3.85 | 23.71$\pm$6.55 | 17.41$\pm$4.66 | 11.17$\pm$3.23 | 69.04$\pm$15.54 |
| Commercial service (n=209) | 18.08$\pm$3.35 | 25.55$\pm$6.58 | 19.08$\pm$4.50 | 12.48$\pm$2.97 | 75.19$\pm$13.89 |
| Enterprise/Institution employee (n=33) | 18.64$\pm$3.00 | 24.94$\pm$7.16 | 18.55$\pm$4.04 | 11.64$\pm$3.82 | 73.76$\pm$13.93 |
| Retired/Unemployed (n=94) | 17.84$\pm$4.20 | 22.99$\pm$6.80 | 17.40$\pm$4.49 | 11.84$\pm$3.08 | 70.09$\pm$15.10 |
| F | 6.665 | 4.506 | 6.116 | 6.836 | 7.311 |
| P | <0.001 | 0.004 | <0.001 | <0.001 | <0.001 |
| Annual Household Income |  |  |  |  |  |
| <30,000 CNY (n=256) | 16.64$\pm$4.38 | 22.89$\pm$7.20 | 17.33$\pm$5.22 | 11.08$\pm$3.48 | 67.95$\pm$17.32 |
| ≥30,000 CNY (n=349) | 18.10$\pm$3.08 | 25.33$\pm$6.10 | 18.58$\pm$4.01 | 12.25$\pm$2.88 | 74.26$\pm$12.54 |
| t | 23.082 | 20.214 | 10.970 | 20.453 | 27.020 |
| P | <0.001 | <0.001 | 0.001 | <0.001 | <0.001 |
| Income Source |  |  |  |  |  |
| Salary(n=361) | 17.93$\pm$3.54 | 24.65$\pm$6.77 | 18.17$\pm$4.64 | 11.92$\pm$3.13 | 72.66$\pm$14.66 |
| Non-Salary(n=244) | 16.82$\pm$3.96 | 23.79$\pm$6.56 | 17.87$\pm$4.55 | 11.51$\pm$3.28 | 69.99$\pm$15.535 |
| t | 12.795 | 2.378 | 0.630 | 2.417 | 4.601 |
| P | <0.001 | 0.124 | 0.427 | 0.121 | 0.032 |
| Disease Stage |  |  |  |  |  |
| HIV(n=442) | 17.61$\pm$3.72 | 24.77$\pm$6.59 | 18.34$\pm$4.65 | 11.85$\pm$3.28 | 72.58$\pm$15.13 |
| AIDS(n=163) | 17.13$\pm$3.81 | 23.02$\pm$6.81 | 17.25$\pm$4.38 | 11.50$\pm$2.94 | 68.90$\pm$14.61 |
| t | 1.969 | 8.289 | 6.775 | 1.440 | 7.183 |
| P | 0.161 | 0.004 | 0.009 | 0.231 | 0.008 |
| Health Insurance Status |  |  |  |  |  |
| No(n=45) | 17.62$\pm$3.85 | 23.71$\pm$7.02 | 17.84$\pm$4.77 | 12.13$\pm$3.04 | 71.31$\pm$15.52 |
| Yes(n=560) | 17.47$\pm$3.75 | 24.35$\pm$6.67 | 18.07$\pm$4.59 | 11.72$\pm$3.21 | 71.61$\pm$15.04 |
| t | 0.069 | 0.377 | 0.096 | 0.685 | 0.016 |
| P | 0.793 | 0.539 | 0.756 | 0.408 | 0.899 |
| Socioeconomic Status |  |  |  |  |  |
| Middle-Lower Class and Below | 17.12$\pm$3.82 | 24.11$\pm$6.45 | 17.69$\pm$4.40 | 11.49$\pm$3.23 | 70.41$\pm$14.84 |
| Middle or Above | 18.24$\pm$3.49 | 24.70$\pm$7.17 | 18.79$\pm$4.92 | 12.30$\pm$3.07 | 74.02$\pm$15.27 |
| t | 12.132 | 1.015 | 7.580 | 8.619 | 7.717 |
| P | 0.001 | 0.314 | 0.006 | 0.003 | 0.006 |
| Happiness in Life |  |  |  |  |  |
| Unhappy(n=71) | 16.41$\pm$4.62 | 22.31$\pm$7.29 | 16.30$\pm$4.90 | 10.82$\pm$3.97 | 65.83$\pm$17.69 |
| Indifferent  （n=280) | 17.17$\pm$3.35 | 24.51$\pm$6.01 | 18.06$\pm$3.92 | 11.90$\pm$2.93 | 71.65$\pm$13.10 |
| Happy(n=254) | 18.12$\pm$3.81 | 24.62$\pm$7.16 | 18.52$\pm$5.08 | 11.85$\pm$3.21 | 73.12$\pm$15.94 |
| t | 7.731 | 3.607 | 6.628 | 3.500 | 6.618 |
| P | <0.001 | 0.028 | 0.001 | 0.031 | 0.001 |
| AIDS Knowledge Awareness |  |  |  |  |  |
| Unaware(n=38) | 13.53$\pm$3.59 | 20.42$\pm$5.73 | 15.39$\pm$3.56 | 9.95$\pm$3.11 | 59.29$\pm$14.50 |
| Aware(n=567) | 17.75$\pm$3.61 | 24.56$\pm$6.68 | 18.23$\pm$4.61 | 11.87$\pm$3.17 | 72.41$\pm$14.75 |
| t | 48.606 | 13.915 | 13.783 | 13.204 | 28.226 |
| P | <0.001 | <0.001 | <0.001 | <0.001 | <0.001 |

Table S3. Analysis of the Impact of Different Variable Characteristics on the Quality of Life Scores of HIV/AIDS Patients in Various Domains($\bar{\text{x}}\text{±}\text{s}$)

| Variable | Physical Domain | Psychological Domain | Independence Domain | Social Relationship Domain | Environmental Domain | Spiritual Domain | Quality of Life |
| --- | --- | --- | --- | --- | --- | --- | --- |
| Gender |  |  |  |  |  |  |  |
| Male (n=538) | 13.91$\text{±}$2.76 | 13.23$\text{±}$2.48 | 13.09$\text{±}$2.13 | 12.49$\text{±}$2.44 | 13.12$\text{±}$2.24 | 13.68$\text{±}$3.10 | 79.53$\text{±}$12.33 |
| Female (n=67) | 13.28$\text{±}$3.04 | 12.53$\text{±}$2.23 | 12.46$\text{±}$2.15 | 12.36$\text{±}$2.30 | 12.51$\text{±}$2.17 | 13.18$\text{±}$2.85 | 76.32$\text{±}$11.26 |
| t | 3.023 | 4.937 | 5.197 | 0.187 | 4.296 | 1.612 | 4.104 |
| P | 0.083 | 0.027 | 0.023 | 0.665 | 0.039 | 0.205 | 0.043 |
| Marital Status |  |  |  |  |  |  |  |
| Married (n=280) | 13.84$\text{±}$2.78 | 13.14$\text{±}$2.48 | 12.92$\text{±}$2.03 | 12.42$\text{±}$2.26 | 12.91$\text{±}$2.08 | 13.55$\text{±}$3.08 | 78.78$\text{±}$11.92 |
| Unmarried (n=325) | 13.85$\text{±}$2.82 | 13.17$\text{±}$2.46 | 13.11$\text{±}$2.23 | 12.53$\text{±}$2.57 | 13.17$\text{±}$2.37 | 13.69$\text{±}$3.07 | 79.52$\text{±}$12.55 |
| t | 0.004 | 0.018 | 1.261 | 0.296 | 1.929 | 0.307 | 0.543 |
| P | 0.953 | 0.894 | 0.262 | 0.586 | 0.165 | 0.580 | 0.462 |
| Education Level |  |  |  |  |  |  |  |
| Junior high or below (n=238) | 13.48$\text{±}$2.83 | 12.82$\text{±}$2.40 | 12.75$\text{±}$2.13 | 12.05$\text{±}$2.30 | 12.46$\text{±}$2.02 | 13.35$\text{±}$2.99 | 76.91$\text{±}$11.60 |
| High school or above (n=367) | 14.08$\text{±}$2.76 | 13.37$\text{±}$2.48 | 13.20$\text{±}$2.14 | 12.75$\text{±}$2.47 | 14.45$\text{±}$2.30 | 13.81$\text{±}$3.11 | 80.65$\text{±}$12.46 |
| t | 6.706 | 7.435 | 6.541 | 12.241 | 28.277 | 3.259 | 13.741 |
| P | 0.010 | 0.007 | 0.011 | 0.001 | <0.001 | 0.072 | <0.001 |
| Occupation |  |  |  |  |  |  |  |
| Farmer (n=269) | 13.45$\text{±}$2.82 | 12.73$\text{±}$2.44 | 12.61$\text{±}$2.15 | 12.10$\text{±}$2.33 | 12.49$\text{±}$2.09 | 13.08$\text{±}$3.11 | 76.45$\text{±}$12.02 |
| Commercial service (n=209) | 14.02$\text{±}$2.67 | 13.54$\text{±}$2.28 | 13.36$\text{±}$2.09 | 12.82$\text{±}$2.38 | 13.38$\text{±}$2.18 | 13.94$\text{±}$2.85 | 81.07$\text{±}$11.60 |
| Enterprise/Institution employee (n=33) | 14.88$\text{±}$2.62 | 14.13$\text{±}$2.77 | 13.97$\text{±}$1.79 | 13.03$\text{±}$2.72 | 14.21$\text{±}$2.15 | 14.36$\text{±}$3.70 | 84.59$\text{±}$12.70 |
| Retired/Unemployed (n=94) | 14.21$\text{±}$2.94 | 13.17$\text{±}$2.62 | 13.14$\text{±}$2.12 | 12.61$\text{±}$2.58 | 13.49$\text{±}$2.58 | 14.23$\text{±}$2.96 | 80.86$\text{±}$12.64 |
| F | 4.168 | 6.340 | 7.597 | 4.306 | 11.919 | 5.542 | 9.181 |
| P | 0.006 | <0.001 | <0.001 | <0.001 | <0.001 | <0.001 | <0.001 |
| Annual Household Income |  |  |  |  |  |  |  |
| <30,000 CNY (n=256) | 13.17$\text{±}$3.11 | 12.91$\text{±}$2.80 | 12.91$\text{±}$2.41 | 12.08$\text{±}$2.88 | 12.69$\text{±}$2.55 | 13.08$\text{±}$3.47 | 76.85$\text{±}$14.11 |
| ≥30,000 CNY (n=349) | 14.34$\text{±}$2.43 | 13.33$\text{±}$2.17 | 13.10$\text{±}$1.92 | 12.77$\text{±}$1.99 | 13.32$\text{±}$1.94 | 14.03$\text{±}$2.68 | 80.88$\text{±}$10.39 |
| t | 26.627 | 4.296 | 1.152 | 12.114 | 11.860 | 14.344 | 16.434 |
| P | <0.001 | 0.039 | 0.284 | 0.01 | 0.01 | <0.001 | <0.001 |
| Income Source |  |  |  |  |  |  |  |
| Salary (n=361) | 14.24$\text{±}$2.60 | 13.34$\text{±}$2.40 | 13.19$\text{±}$2.02 | 12.66$\text{±}$2.32 | 13.29$\text{±}$2.12 | 13.90$\text{±}$2.96 | 80.62$\text{±}$11.62 |
| Non-salary (n=244) | 13.26$\text{±}$2.98 | 12.88$\text{±}$2.53 | 12.78$\text{±}$2.30 | 12.21$\text{±}$2.56 | 12.69$\text{±}$2.37 | 13.23$\text{±}$3.19 | 77.04$\text{±}$12.86 |
| t | 18.386 | 5.149 | 5.187 | 5.109 | 10.730 | 7.103 | 12.640 |
| P | <0.001 | 0.024 | 0.023 | 0.024 | 0.001 | 0.008 | <0.001 |
| Disease Stage |  |  |  |  |  |  |  |
| HIV(n=442) | 14.01$\text{±}$2.71 | 13.26$\text{±}$2.43 | 13.10$\text{±}$2.18 | 12.59$\text{±}$2.49 | 13.15$\text{±}$2.28 | 13.77$\text{±}$3.07 | 79.88$\text{±}$12.26 |
| AIDS(n=163) | 13.39$\text{±}$2.99 | 12.87$\text{±}$2.55 | 12.82$\text{±}$2.02 | 12.18$\text{±}$2.24 | 12.77$\text{±}$2.12 | 13.25$\text{±}$3.05 | 77.27$\text{±}$12.06 |
| t | 5.829 | 3.006 | 2.092 | 3.456 | 3.543 | 3.480 | 5.455 |
| P | 0.016 | 0.083 | 0.149 | 0.064 | 0.060 | 0.063 | 0.020 |
| CD4+ T lymphocyte levels（cells/μL) |  |  |  |  |  |  |  |
| <200(n=86) | 13.24$\text{±}$2.60 | 12.81$\text{±}$2.42 | 12.92$\text{±}$2.25 | 12.17$\text{±}$2.51 | 12.97$\text{±}$2.55 | 13.06$\text{±}$2.87 | 77.18$\text{±}$12.28 |
| 200-350(n=117) | 13.54$\text{±}$2.76 | 13.02$\text{±}$2.59 | 12.70$\text{±}$2.21 | 11.95$\text{±}$2.24 | 12.82$\text{±}$2.12 | 13.59$\text{±}$3.28 | 77.61$\text{±}$12.41 |
| >350(n=402) | 14.06$\text{±}$2.83 | 13.27$\text{±}$2.43 | 13.14$\text{±}$2.09 | 12.70$\text{±}$2.44 | 13.13$\text{±}$2.20 | 13.76$\text{±}$3.05 | 80.06$\text{±}$12.14 |
| F | 3.908 | 1.446 | 2.026 | 5.194 | 0.966 | 1.874 | 3.166 |
| P | 0.021 | 0.236 | 0.133 | 0.006 | 0.381 | 0.154 | 0.043 |
| Presence of AIDS-related Symptoms |  |  |  |  |  |  |  |
| No（n=574) | 13.91$\text{±}$2.73 | 13.21$\text{±}$2.41 | 13.06$\text{±}$2.09 | 12.52$\text{±}$2.40 | 13.11$\text{±}$2.21 | 13.69$\text{±}$3.07 | 79.50$\text{±}$12.01 |
| Yes(n=31) | 12.61$\text{±}$3.75 | 12.15$\text{±}$3.26 | 12.42$\text{±}$2.91 | 11.65$\text{±}$2.84 | 11.90$\text{±}$2.49 | 12.52$\text{±}$2.98 | 73.25$\text{±}$15.15 |
| t | 6.375 | 5.411 | 2.605 | 3.879 | 8.640 | 4.308 | 7.723 |
| P | 0.012 | 0.020 | 0.107 | 0.049 | 0.003 | 0.038 | 0.006 |
| Health Insurance Status |  |  |  |  |  |  |  |
| No(n=45) | 13.13$\text{±}$3.25 | 12.85$\text{±}$2.89 | 12.71$\text{±}$2.07 | 12.00$\text{±}$3.09 | 12.87$\text{±}$2.75 | 12.76$\text{±}$3.54 | 76.32$\text{±}$14.90 |
| Yes(n=560) | 13.90$\text{±}$2.75 | 13.18$\text{±}$2.43 | 13.05$\text{±}$2.15 | 12.52$\text{±}$2.37 | 13.06$\text{±}$2.20 | 13.70$\text{±}$3.02 | 79.41$\text{±}$12.00 |
| t | 3.140 | 0.725 | 1.032 | 1.900 | 0.320 | 3.944 | 2.649 |
| P | 0.077 | 0.395 | 0.310 | 0.169 | 0.572 | 0.047 | 0.104 |
| Socioeconomic Status |  |  |  |  |  |  |  |
| Middle-Lower Class and Below | 13.30$\text{±}$2.28 | 12.59$\text{±}$2.33 | 12.62$\text{±}$2.11 | 11.96$\text{±}$2.26 | 12.49$\text{±}$2.03 | 13.12$\text{±}$3.10 | 76.07$\text{±}$11.51 |
| Middle or Above | 14.97$\text{±}$2.50 | 14.33$\text{±}$2.32 | 13.86$\text{±}$1.97 | 13.55$\text{±}$2.42 | 14.21$\text{±}$2.21 | 14.68$\text{±}$2.72 | 85.61$\text{±}$11.22 |
| t | 51.330 | 74.082 | 48.459 | 63.094 | 90.497 | 36.296 | 92.605 |
| P | <0.001 | <0.001 | <0.001 | <0.001 | <0.001 | <0.001 | <0.001 |
| Happiness in Life |  |  |  |  |  |  |  |
| Unhappy(n=71) | 11.93$\text{±}$3.29 | 11.32$\text{±}$3.05 | 12.03$\text{±}$2.60 | 10.51$\text{±}$2.76 | 11.32$\text{±}$2.54 | 11.49$\text{±}$3.95 | 68.60$\text{±}$14.26 |
| Indifferent  （n=280) | 13.29$\text{±}$2.47 | 12.59$\text{±}$1.92 | 12.60$\text{±}$1.79 | 11.99$\text{±}$1.91 | 12.44$\text{±}$1.66 | 13.07$\text{±}$2.49 | 75.98$\text{±}$8.97 |
| Happy(n=254) | 14.99$\text{±}$2.51 | 14.29$\text{±}$2.30 | 13.76$\text{±}$2.13 | 13.57$\text{±}$2.32 | 14.21$\text{±}$2.14 | 14.84$\text{±}$2.88 | 85.66$\text{±}$11.34 |
| t | 50.492 | 65.705 | 31.039 | 66.804 | 83.620 | 47.951 | 93.519 |
| P | <0.001 | <0.001 | <0.001 | <0.001 | <0.001 | <0.001 | <0.001 |
| AIDS Knowledge Awareness |  |  |  |  |  |  |  |
| Unaware(n=38) | 11.76$\text{±}$3.34 | 11.94$\text{±}$2.68 | 11.95$\text{±}$2.46 | 10.82$\text{±}$3.06 | 11.34$\text{±}$2.63 | 11.84$\text{±}$3.82 | 69.65$\text{±}$15.38 |
| Aware(n=567) | 13.98$\text{±}$2.70 | 13.24$\text{±}$2.43 | 13.10$\text{±}$2.10 | 12.59$\text{±}$2.34 | 13.16$\text{±}$2.17 | 13.75$\text{±}$2.98 | 79.82$\text{±}$11.76 |
| t | 23.241 | 10.039 | 10.392 | 19.642 | 24.412 | 14.012 | 25.513 |
| P | <0.001 | 0.002 | 0.001 | <0.001 | <0.001 | <0.001 | <0.001 |
